# Prevalence and comorbidity of diabetes mellitus among non-institutionalized older adults in Germany - results of the national telephone health interview survey ‘German Health Update (GEDA)’ 2009

**DOI:** 10.1186/1471-2458-13-166

**Published:** 2013-02-23

**Authors:** Yong Du, Christin Heidemann, Antje Gößwald, Patrick Schmich, Christa Scheidt-Nave

**Affiliations:** 1 Department of Epidemiology and Health Monitoring, Division of Non-Communicable Disease Epidemiology, Robert Koch Institute, General-Pape-Str. 62-66, D-12101, Berlin, Germany

**Keywords:** Diabetes, Prevalence, Comorbidity, Germany

## Abstract

**Background:**

Despite the major public health impact of diabetes, recent population-based data regarding its prevalence and comorbidity are sparse.

**Methods:**

The prevalence and comorbidity of diabetes mellitus were analyzed in a nationally representative sample (N = 9133) of the non-institutionalized German adult population aged 50 years and older. Information on physician-diagnosed diabetes and 20 other chronic health conditions was collected as part of the national telephone health interview survey ‘German Health Update (GEDA)’ 2009. Overall, 51.2% of contacted persons participated. Among persons with diabetes, diabetes severity was defined according to the type and number of diabetes-concordant conditions: no diabetes-concordant condition (grade 1); hypertension and/or hyperlipidemia only (grade 2); one comorbidity likely to represent diabetes-related micro- or macrovascular end-organ damage (grade 3); several such comorbidities (grade 4). Determinants of diabetes severity were analyzed by multivariable ordinal regression.

**Results:**

The 12-month prevalence of diabetes was 13.6% with no significant difference between men and women. Persons with diabetes had a significantly higher prevalence and average number of diabetes-concordant as well as diabetes-discordant comorbidities than persons without diabetes. Among persons with diabetes, 10.2%, 46.8%, 35.6% and 7.4% were classified as having severity grade 1–4, respectively. Determinants of diabetes severity included age (cumulative odds ratio 1.05, 95% confidence interval 1.03-1.07, per year) and number of discordant comorbidities (1.40, 1.25-1.55). With respect to specific discordant comorbidities, diabetes severity was correlated to depression (2.15, 1.29-3.56), respiratory disease (2.75, 1.72-4.41), musculoskeletal disease (1.53, 1.06-2.21), and severe hearing impairment (3.00, 1.21-7.41).

**Conclusions:**

Diabetes is highly prevalent in the non-institutionalized German adult population 50 years and older. Diabetes comorbidities including diabetes-concordant and diabetes-discordant conditions need to be considered in epidemiological studies, in order to monitor disease burden and quality of diabetes care. Definitional standards of diabetes severity need to be refined and consented.

## Background

Diabetes mellitus is a highly common metabolic disorder with major public health impact due to its detrimental consequences causing severe end-organ damage, including cardiovascular and neurological complications, diabetic retinopathy, and diabetic nephropathy [1-3]. Diabetes prevalence has dramatically increased in many countries over the past decades [4,5]. However, many countries lack epidemiological studies that permit surveillance of diabetes prevalence at the national level. Beyond that, population-based data on diabetes comorbidity are needed to provide insight into the burden of disease, to define subgroups with specific health care needs, and to monitor quality of diabetes care [2,3,6]. Concomitant health conditions are known to affect both the course and outcome of the disease treatment [2,3,7-9]. Thus, guidelines for diabetes mellitus may need specific adjustments given an increasing number of older diabetics with multiple health conditions [10].

The prevalence of diabetes among adults in Germany was estimated in several previous national health surveys [11] as well as in 5 regional population-based studies [12] conducted between 1990 and 2005. At the national level, the prevalence of diabetes among adults 25–65 years ranged between 4.6-5.2% in the earliest and 5.3% in the latest survey and no significant increase over time was observed [11]. Diabetes prevalence was found to vary considerably between regions, with the highest prevalence estimates found in Northeastern Germany and the lowest estimates in the South [12]. In addition, diabetes prevalence among adults in Germany has been estimated based on statutory health insurance claim data [13-16] or data collected in the general practice setting [17,18]. These studies consistently show higher prevalence estimates compared to results from population-based studies. A statistically significant increase in prevalence over time was evident from periodically repeated analyses of data from a large sickness fund in the federal state of Hesse [13-15]. No study in Germany has systematically analyzed diabetes comorbidity so far. The prevalence and patterns of morbidity and multimorbidity among older adults in Germany were analyzed in previous studies based on data from the 2009 national health telephone survey [19] or insurance claims [20] or general practices [21]. Detailed information on diabetes comorbidity was collected in general practice studies, but only the proportions of patients with diabetes-specific complications were reported. Very consistently, nearly half of adults with diabetes already had either macro- or microvascular complications [17,18]. Against this background, we used data of the national health interview survey ‘German Health Update (GEDA)’ 2009 to determine the prevalence of known diabetes and to analyze patterns of diabetes comorbidity including diabetes-related as well as other comorbidities in the non-institutionalized population 50 years and older.

## Methods

### Study design and study population

The national telephone health interview survey *Gesundheit in Deutschland Aktuell* 2009 (German Health Update, GEDA 2009) was conducted by the Robert Koch Institute between July 2008 and June 2009 [22]. In GEDA 2009, a two-stage sampling procedure was applied. First, a pool of about 223,000 telephone numbers from complete listings of landline telephone extensions was randomly generated, applying the Gabler-Häder method [23]. This method assures that non-private household phone numbers such as those for public service use, fax numbers as well as multiple numbers within household are excluded, while households with unregistered telephone extensions are included in the ‘target sample’. Secondly, random sampling at the individual level was achieved by the ‘last-birthday-method’, i.e. the adult household member whose birthday most closely preceded the date of first contact to the respective household was included in the target sample of contact persons [23]. Altogether, 21,262 persons (9,148 men and 12,114 women) of contacted persons 18 years of age and older completed the survey. This corresponds to a cooperation rate at the individual level of 51.2%, which represents the cooperation rate at the respondent level. (Respondent-level cooperation rates are calculated using contacts with and refusals from known respondents) [24]. Telephone interviews were conducted between 10 a.m. and 8.30 p.m., six days a week. Interviews took an average of 30 minutes. The study was approved by The Federal Commissioner for Data Protection and Freedom of Information. Verbal informed consent was provided by all participants prior to the interview.

The present study was confined to 9,155 adults aged 50 years and older. Among these, 22 persons did not answer the question concerning a medical history of diabetes and were hence excluded. Thus, the final study population consisted of 9,133 adults (3,855 men and 5,278 women).

### Data collection and definition of study variables

Using a standardized computer-assisted interview (CATI) technique, GEDA 2009 collected information regarding socio-demographic characteristics, health-related behavior and medical history of diabetes and 20 other chronic health conditions. Participants were considered to suffer from diabetes if they answered ‘yes’ to the following sequence of questions: ‘Has a doctor ever told you that you have diabetes?’ and ‘Have you been suffering from diabetes during the past 12 months?’. Information regarding the type of diabetes or use of antidiabetic medication was not collected. The same criteria as for diabetes were applied to the definition of any other of the following health conditions: hyperlipidemia, hypertension, chronic heart failure, asthma, chronic bronchitis, chronic renal disease, chronic liver disease, gastritis or duodenitis, gastric or duodenal ulcer, osteoarthritis, rheumatoid arthritis, osteoporosis, depression and chronic back pain lasting for at least three months. For the definition of hypertension and hyperlipidemia, information on current blood pressure or lipid-lowering medication was available and considered. A history of cancer, stroke, myocardial infarction (MI) or other coronary heart disease (CHD) such as angina pectoris (AP) was defined if participants reported a lifetime medical history, e. g. that these conditions had ever been diagnosed by a doctor. Assessment of visual or hearing impairment was based on the European Health Status Module questions [25]. Participants were asked if they had any difficulties either in reading printed newspaper or in identifying the face of a person 4 meters away, using glasses or any other reading aid as necessary. Hearing impairment was measured by asking participants if they had any difficulties to follow a conversation with several people, using a hearing aid as necessary. Possible answer choices for the questions were: (1) can read/hear clearly without difficulty, (2) can read/hear with some difficulty, (3) can read/hear with great difficulty and (4) unable to read/hear at all. Participants in categories ‘3’ or ‘4’ were defined as experiencing ‘severe visual/hearing impairment’.

Comorbidities were differentiated as diabetes-concordant or diabetes-discordant based on current evidence in support of a pathophysiological link to diabetes [26]. Concordant comorbidities included metabolic risk factors (hypertension and hyperlipidemia) known to be components of the metabolic syndrome as well as conditions likely to represent major complications of diabetes, such as cardiovascular end-organ disease (CVD), chronic renal disease, and severe visual impairment. Chronic health conditions other than these were considered as discordant comorbidities. Individual conditions, whenever possible, were further grouped according to organ systems (e. g. heart, cardiovascular, respiratory, digestive musculoskeletal).

Among persons with known diabetes, disease severity was graded according to the type and number of diabetes-concordant comorbidities along a four-point scale: no diabetes-concordant comorbidity (grade 1); hypertension and/or hyperlipidemia only (grade 2); one comorbidity likely to represent diabetes-related complications (grade 3), and several such comorbidities (grade 4). Grade 3 and 4 diabetes severity levels were assigned irrespective of cardiometabolic conditions.

Body mass index (BMI) was computed from self-reported measures of body weight and height and categorized as ‘normal’, ‘overweight’ and ‘obese’ according to WHO criteria [27]. Smoking status was defined based on answers to the question ‚Do you smoke – even occasionally?’ Study participants who answered ‘yes, daily’ or ‘yes, occasionally’ were defined as current smokers, while those who answered ‘no, I quit smoking’ or ‘no, I never smoked’ were categorized as ‘ex-smokers’ or ‘non-smokers’, respectively. Sports activity was defined by the question ’Did you engage in any sports activities in the past three months?’ Response choices were ‘no’, ‘yes, less than 2 hours/week’, ‘2-4 hours/week’ and ‘more than 4 hours/week’. Educational attainment was defined by internationally used CASMIN (**C**omparative **A**nalysis of **S**ocial **M**obility in **I**ndustrial **N**ations) classification [28]. Considering geographic differences in diabetes prevalence in Germany [12], regional area of residence was defined as follows: northern Germany (federal states: *Bremen*, *Hamburg*, *Niedersachsen* and *Schleswig-Holstein*); central Germany (*Hessen*, *Nordrhein-Westfalen, Rheinland-Pfalz* and *Saarland);* southern Germany (*Baden-Württemberg and Bayern)*; eastern Germany (*Berlin*, *Brandenburg, Mecklenburg-Vorpommern, Sachsen, Sachsen-Anhalt* and *Thüringen*)*.* All federal states in ‘eastern Germany’ excluding Berlin represent the former German Democratic Republic (East Germany), while the others represent the former Federal Republic of Germany (West Germany).

### Statistical methods

SPSS software (version 18.0.3, SPSS Inc. Chicago, IL) was used for statistical analyses. Descriptive statistics were used to assess the 12-month prevalence of known diabetes according to socio-demographic and behavioral characteristics and to assess the prevalence of comorbidities among persons with and without diabetes. The Mann–Whitney-U test and general linear model procedures were used to compare the median and mean number of comorbidities between persons with and without diabetes. The second-order Rao-Scott chi-square test provided by the SPSS complex sample procedure was used to test for group differences in categorical variables. Odds ratios and 95% confidence intervals (95% CI) as a measure of association between diabetes and other chronic health conditions were obtained from logistic regression models adjusting for age, sex, region of residence, BMI, smoking status, sports activities, and educational attainment. In order to identify determinants of diabetes severity, we fitted ordinal regression models using the grade of diabetes severity with four possible response levels (grades 1–4) for the dependent variable. Two separate models were fitted including the number (model 1) or the type (model 2) of additional, i. e. diabetes-discordant comorbidities. Sex-specific analyses were performed. Results were presented for men and women combined, unless there was evidence for a sex difference based on formal testing for interaction with sex. All reported results are weighted. Weights used in the analysis represent a combination of design weights (adjusting for sampling design) and adjustment weights (correcting deviations between the study population and German population statistics of December 31, 2007 within strata of age, sex, educational attainment, and residential region) [22]. The SPSS complex samples procedure was applied throughout analyses to keep statistical inferences as conservative as possible. Statistical significance was set at Alpha < 0.05 based on two-sided tests.

## Results

### Prevalence of known diabetes by socio-demographic and behavioral characteristics

Among 9,133 study participants, a total of 1,035 persons (488 men, 547 women) 50–93 years of age classified as having known diabetes. The overall weighted 12-month prevalence was 13.6% with no significant difference between men and women (Table 1). The prevalence of diabetes significantly and positively correlated with age and BMI, whereas a significant and inverse association existed with educational attainment and sports activity. Diabetes prevalence was higher among ex-smokers and non-smokers compared to current smokers. Regional differences were observed, with significantly higher prevalence estimates in eastern compared to northern, central or southern federal states. Except for smoking status, characteristics shown in Table 1 were significantly and independently associated with diabetes in multivariable logistic regression models. Results persisted in sex- specific analyses (data not shown).

**Table 1:** 12-month prevalence of known diabetes mellitus among German adults aged 50 years and older (N = 9133) by socio-demographic and behavioral characteristics

**Characteristics**	**Prevalence % (95% CI)**	**Unadjusted OR (95% CI)**	**Adjusted OR (95% CI)**
Overall	**13.6 (12.7-14.7)**		
**Sex**			
Men	13.9 (12.5-15.5)	1.04 (0.88-1.24)	1.02 (0.83-1.25)
Women	13.4 (12.1-14.9)		1.00
Age groups ^***^(yrs)			
50-54	6.5 (5.1-8.2)	1.00	1.00
55-59	9.5 (7.9-11.5)	1.51 (1.09-2.09)	1.48 (1.05-2.07)
60-64	11.8 (9.6-14.5)	1.93 (1.37-2.71)	1.96 (1.38-2.78)
65-69	14.2 (11.9-16.8)	2.37 (1.72-3.26)	2.20 (1.56-3.11)
70-74	20.1 (17.1-23.4)	3.60 (2.62-4.96)	3.59 (2.54-5.06)
75+	19.4 (16.5-22.6)	3.46 (2.52-4.75)	3.55 (2.49-5.05)
Region^**^			
East	17.1 (15.0-19.6)	1.00	1.00
North	13.3 (10.9-16.1)	0.74 (0.56-0.98)	0.73 (0.54-0.98)
Central	13.0 (11.4-14.9)	0.72 (0.58-0.90)	0.71 (0.56-0.91)
South	11.9 (10.1-13.9)	0.65 (0.51-0.83)	0.60 (0.46-0.78)
BMI^*** ^**(kg/m**^**2**^**)**			
< 25 (Normal)^#^	5.9 (4.9-7.2)	1.00	1.00
≥ 25 (Overweight)	12.5 (11.1-14.2)	2.22 (1.73-2.85)	2.10 (1.63-2.71)
≥ 30 (Obese)	28.7 (25.9-31.8)	6.65 (5.17-8.55)	5.74 (4.42-7.46)
Smoking status^***^			
Current smoker	9.5 (7.9-11.6)	1.00	1.00
Ex smoker	14.9 (13.2-16.7)	1.66 (1.28-2.14)	1.15 (0.87-1.52)
Non-smoker	14.5 (13.0-16.2)	1.61 (1.25-2.07)	1.01 (0.76-1.35)
Sports activity in the past 3 months^***^			
No sports	18.4 (16.7-20.3)	2.44 (1.89-3.15)	1.70 (1.30-2.23)
<4 hours/week	10.6 (9.2-12.1)	1.28 (0.97-1.68)	1.14 (0.86-1.53)
over 4 hours/week	8.4 (6.9-10.4)	1.00	1.00
Educational attainment^***^			
Primary	16.6 (15.0-18.4)	1.99 (1.63-2.43)	1.28 (1.02-1.60)
Middle	11.2 (9.9-12.8)	1.26 (1.02-1.57)	1.04 (0.82-1.32)
High	9.1 (7.9-10.5)	1.00	1.00

National Health Telephone Interview Survey “German Health Update (GEDA)” 2009).

Estimates of 12-month prevalence (%) and their 95% confidence intervals (95% CI) were weighted according to German population statistics (December 31, 2007).

^#^ including 101 (1%) under-weighted subjects whose body mass index (BMI) is less than 18.5 kg/m^2^.

^*^ p < .05, ^**^ p < .01, ^***^ p < .001, derived from Rao-Scott chi-square tests for the difference of prevalence estimates within specific group.

Adjusted and unadjusted odds ratio (OR) and 95% confidence intervals (95% CI) were derived from logistic regression models. Variables in the adjusted logistic regression model include all variables listed in Table 1.

### Association between diabetes and other chronic health conditions

Table 2 compares the prevalence of comorbidities between persons with and without diabetes according to individual conditions as well as disease categories. In both groups, cardiometabolic risk factors were the most prevalent health conditions, followed by osteoarthritis and chronic back pain.

**Table 2:** Prevalence of chronic health conditions and association with known diabetes mellitus among German adults aged 50 years and older (N = 9133)

**Health conditions**		**Diabetics (N = 1035)**	**Non-Diabetics (N = 8098)**	**Model 1**		**Model 2**	
		**Prevalence (%)**	**Prevalence (% )**	**OR**^**1**^	**95% CI**	**OR**^**2**^	**95% CI**
**Diabetes-concordant conditions (No. 1–8)**		89.8^***^	60.4	4.99^***^	3.76-6.62	3.71^***^	2.76-4.99
**1**	Hypertension	73.5^***^	40.8	3.60^***^	2.95-4.40	2.57^***^	2.08-3.18
**2**	Hyperlipidemia	52.8^***^	32.4	2.21^***^	1.84-2.64	2.00^***^	1.65-2.43
	***Cardiometabolic risk factors (No .1-2)***	86.3^***^	56.5	4.33^***^	3.38-5.55	3.20^***^	2.47-4.15
**3**	Angina pectoris	28.5^***^	11.6	2.54^***^	2.03-3.18	2.28^***^	1.79-2.90
**4**	Myocardial infarction	14.0^***^	5.3	2.50^***^	1.86-3.37	2.30^***^	1.66-3.19
	***Coronary Heart Disease (CHD) (No. 3–4)***	31.0^***^	13.0	2.52^***^	2.03-3.13	2.24^***^	1.77-2.84
**5**	Congestive heart failure	10.9^***^	4.9	1.97^***^	1.43-2.71	1.81^***^	1.28-2.57
	***Diseases of the heart (No.3-5)***	34.0^***^	15.2	2.38^***^	1.93-2.94	2.11^***^	1.68-2.65
**6**	Stroke	7.9^***^	3.8	1.80^**^	1.25-2.59	1.69^*^	1.13-2.54
	***Cardiovascular Disease (CVD) (No. 3–6)***	37.2^***^	17.3	2.35^***^	1.91-2.87	2.10^***^	1.69-2.62
**7**	**Chronic renal disease**	8.3^***^	1.8	4.33^***^	2.79-6.72	3.72^***^	2.30-6.03
**8**	**Severe visual impairment**	6.0^*^	4.1	1.23	0.85-1.78	1.16	0.79-1.71
					-		-
**Diabetes-discordant conditions (No. 9–20)**		68.5^***^	58.3	1.40^***^	1.16-1.69	1.23^*^	1.00-1.50
**9**	Asthma	8.9^**^	5.9	1.47^*^	1.07-2.02	1.28	0.90-1.80
**10**	Chronic bronchitis	10.8^**^	7.0	1.50^**^	1.12-2.01	1.30	0.96-1.78
	***Lower respiratory diseases (No.9-10)***	14.4^***^	9.6	1.48^**^	1.15-1.91	1.30	0.99-1.71
**11**	Gastritis/duodenitis	6.3	4.6	1.52^*^	1.07-2.17	1.51^*^	1.05-2.16
**12**	Gastricduodenal ulcers	1.9^*^	0.7	2.57^*^	1.21-5.48	2.26^*^	1.10-4.63
	***Digestive disorders (No. 11–12)***	7.3^*^	5.0	1.60^**^	1.13-2.25	1.58^*^	1.12-2.24
**13**	Osteoarthritis	42.3^***^	31.4	1.50^***^	1.25-1.81	1.27^*^	1.04-1.54
**14**	Rheumatoid arthritis	14.1^***^	7.4	2.01^***^	1.52-2.65	1.65^***^	1.22-2.23
**15**	Osteoporosis	12.6	9.9	1.13	0.85-1.51	1.10	0.81-1.49
**16**	Chronic back pain	34.5^***^	26.0	1.45^***^	1.20-1.75	1.20	0.98-1.46
	***Musculoskeletal diseases (No.13-16)***	56.5^***^	46.5	1.39^***^	1.16-1.67	1.19	0.98-1.44
**17**	**Chronic liver disease**	5.7^***^	1.8	3.30^***^	2.10-5.18	2.72^***^	1.76-4.19
**18**	**Cancer (any malignancy)**	16.8^***^	11.4	1.37^*^	1.07-1.77	1.43^**^	1.10-1.85
**19**	**Depression**	8.6	7.0	1.44^*^	1.06-1.95	1.24	0.90-1.70
**20**	**Severe hearing impairment**	5.5	5.2	0.86	0.57-1.29	0.72	0.47-1.11

National Telephone Health Interview Survey ‘German Health Update (GEDA)’ 2009.

^*^ p < .05, ^**^ p < .01, ^***^ p < .001, Rao-Scott chi-square test for independence of the associations between diabetes and other chronic conditions.

OR^**1**^: adjusted for age (continuous variable) and sex.

OR^**2**^: adjusted for age (continuous variable), sex, region of residence, body mass index, smoking status, sports activities and educational attainment. ^*^ p < .05; ^**^ p < .01, ^***^ p < .001.

CHD: coronary heart disease; CVD: cardiovascular disease; OR: Odds ratio; 95% CI: 95% confidence intervals.

Adjusting for age and sex, diabetes was significantly associated with all chronic conditions except for osteoporosis, severe visual and hearing impairment (Table 2). Further adjustment for educational attainment, region of residence, BMI, smoking status, and sports activity generally reduced the strength of the associations. Significant associations persisted except for associations of diabetes with lower respiratory disease, chronic back pain, and depression (Table 2). Associations with diabetes were strongest for cardiometabolic and cardiovascular conditions, chronic renal and chronic liver disease. An interaction with sex was observed for the association between diabetes and depression (p = .022). In sex-specific analyses, women, but not men with diabetes were significantly more likely to also have depression (1.57; 95% CI: 1.08-2.30 vs. 0.83; 95% CI: 0.48-1.46).

On average, persons with diabetes had significantly more comorbidities than persons without diabetes (mean ±SD: 3.7±2.4 vs. 2.2±2.0; median: 3 vs. 2, p < .001). Differences in number of comorbidities between the two groups were independent of differences in age, sex, BMI, region of residence, smoking status, sports activities, and education (data not shown). Significant differences between persons with and without diabetes were observed for diabetes-concordant (mean±SD: 2.0±1.3 vs. 1.0±1.1; median: 2 vs. 1, p < .001) as well as diabetes-discordant comorbidities (mean±SD: 1.7±1.7 vs. 1.2±1.4; median: 1 vs. 1, p < .001).

Among persons with diabetes, 94.6% (92.3% of men; 96.7% of women) suffered from at least one comorbid condition and 80.2% (74.0% of men; 85.5% of women) had two or more comorbidities. By comparison, among non-diabetics, 78.5% (74.6% of men; 81.8% of women) had at least one and 56.1% (51.1% of men; 61.4% in women) had at least two additional chronic conditions (data not shown).

### Patterns of diabetes-concordant comorbidity and severity of diabetes among diabetics

Table  3  depicts  patterns  of  diabetes-concordant comorbidities among diabetics. Total as well as mutually exclusive prevalence estimates are shown for each of the possible combinations. For example, a total of 872 diabetics or 86.3% had at least one cardiometabolic condition. Among these, more than half (n = 497, 46.8%) had none of any other concordant comorbidities. Overall, 352 diabetics or 37.2% had CVD. Among these, only a small proportion (n = 25; 2.2%) had CVD alone, while the vast majority also had at least one cardiometabolic condition (n = 265; 27.5%) without any other concordant comorbidities. Smaller proportions of diabetics with CVD showed other comorbidity patterns, e. g. n = 27 (3.4%) had any cardiometabolic condition plus chronic renal disease without severe visual impairment, n = 22 (1.8%) had any cardiometabolic condition plus severe visual impairment without chronic renal disease, and n = 7 (0.5%) had any cardiometabolic condition plus chronic renal disease as well as severe visual impairment. In sex-specific analyses, similar patterns were found for men as for women women (data not shown).

**Table 3:** Prevalence and patterns of diabetes-concordant comorbidity among German adults aged 50 years and older with known diabetes mellitus (N = 1035)

**Diabetes concordant comorbidities**				**Total**		**Mutually exclusive**		Severity of diabetes*
**Cardio-metabolic conditions**	**CVD**	**Chronic renal disease**	**Severe visual impairment**	**N**	**Prevalence of subjects having the indicated comorbidities % (95% CI)**	**N**	**Prevalence of subjects having the indicated comorbidities only % (95% CI)**	
						120	10.2 (8.0-13.0)	Grade 1
								(n = 120)
X				872	86.3 (83.2-88.8)	497	46.8 (42.7-50.9)	Grade 2
								(n = 497)
	X			352	37.2 (33.2-41.3)	25	2.2 (1.3-3.7)	
		X		72	8.3 (6.1-11.2)	4	0.2 (0.1-0.4)	Grade 3
			X	70	6.0 (4.4-8.0)	8	0.8 (0.3-2.0)	(n = 350)
X	X			321	33.4 (29.6-37.5)	265	27.5 (24.0-31.4)	35.6%
X		X		64	7.4 (5.4-10.2)	24	3.2 (1.8-5.4)	(31.7-39.7%)
X			X	59	4.9 (3.5-6.7)	24	2.2 (1.3-3.7)	
	X	X		38	4.7 (3.1-7.1)	3	0.6 (0.2-2.4)	
	X		X	32	2.8 (1.8-4.3)	2	0.3 (0.1-1.1)	
		X	X	14	0.9 (0.5-1.7)	0	0	Grade 4
	X	X	X	8	0.6 (0.3-1.4)	1	0.1 (0.0-0.6)	(n = 68)
X	X		X	29	2.4 (1.5-3.8)	22	1.8 (1.0-3.2)	7.4%
X		X	X	13	0.8 (0.5-1.6)	6	0.3 (0.1-0.7)	(5.4-10.0%)
X	X	X		34	3.9 (2.5-6.1)	27	3.4 (2.0-5.5)	
X	X	X	X	7	0.5 (0.2-1.2)	7	0.5 (0.2-1.2)	

National Telephone Health Interview Survey ‘German Health Update (GEDA)’ 2009.

Cardiometabolic risk factors: hypertension, hyperlipidemia.

CVD: angina pectoris, myocardial infarction, chronic heart failure and stroke.

* Severity of diabetes was defined as: grade 1 = no diabetes-concordant comorbidity; grade 2 = any cardiometabolic condition without further diabetes-concordant comorbidity; grade 3 = any one diabetes-concordant end-organ complications (CVD or chronic renal disease or several visual impairment) irrespective of cardiometalic conditions; grade 4 =two or more diabetes-specific end-organ diseases irrespective of cardiometabolic conditions Grade 3 and 4 are irrespective of cardiometabolic conditions.

Table 3 further summarizes patterns of diabetes-concordant comorbidities with respect to diabetes severity grade 1–4. A total of 120 persons or 10.2% of persons with diabetes had no diabetes-concordant comorbidities corresponding to severity grade 1; 46.8% reported hypertension/hyperlipidemia only (grade 2), and 43% had at least one comorbidity likely to represent major macro- or microvascular end-organ complications (grade 3). The proportion of persons with diabetes who had at least one diabetes-discordant condition continuously and significantly increased with increasing diabetes severity grade, ranging from 47.7% (men: 38.2%; women: 60.2%) among persons with diabetes severity grade 1, to 64.3% (men: 53.7%; women: 72.2%) among those with severity grade 2, to 74.9% (men: 67.3%; women: 83.0%) among those with severity grade 3 and to 93.7% (men: 92.4%; women: 94.2%) among those with severity grade 4 (data not shown in Table 3).

### Independent correlates of diabetes severity among persons with diabetes

In ordinal regression analyses, the grade of diabetes severity among persons with diabetes was significantly and positively related to the total number of diabetes-discordant comorbidities (Table 4, model 1). Except for age [cumulative odds ratio (COR) = 1.05, 95% CI: 1.03-1.07 per year] none of the covariables were independently related to the grade of diabetes severity (data not shown in Table 4). Current smoking (reference category: never smoking) was the only independent variable showing a significant interaction with sex (p = .014). In sex-specific analyses, current smoking was significantly associated with the grade of diabetes severity among women (3.03, 1.37-6.72) but not among men (0.62, 0.25-1.49).

**Table 4:** Association between diabetes-discordant comorbidities and diabetes severity among German adults aged 50 years and older with known diabetes mellitus (N = 1035)

	**COR**^**1**^	**95% CI**	**COR**^**2**^	**95% CI**
***Model 1***				
No. of diabetes-discordant comorbidities	1.38	1.24-1.53	1.40	1.25-1.55
***Model 2***				
**Type of diabetes-discordant comorbidities**				
Lower respiratory disease	2.59	1.66-4.05	2.75	1.72-4.40
Digestive disease	1.34	0.76-2.39	1.42	0.79-2.57
Musculoskeletal diseases	1.54	1.07-2.21	1.53	1.06-2.21
Chronic liver disease	1.79	0.87-3.67	1.86	0.84-4.16
Any cancers	0.67	0.41-1.11	0.63	0.38-1.07
Depression	2.07	1.23-3.48	2.21	1.33-3.67
Severe hearing impairment	2.93	1.16-7.37	2.90	1.17-7.15

National Health Telephone Interview Survey ‘German Health Update (GEDA)’ 2009.

COR = Cumulative odds ratios (COR) as obtained from multivariable ordinal regression analysis with grade of diabetes severity (grade 1–4) as the dependent variable.

COR^1^: adjusted for age (continuous variable) and sex.

COR^2^: adjusted for age (continuous variable), sex, region of residence, body mass index, smoking status, sports activities and educational attainment.

Model 1: with no. of diabetes-discordant comorbidities as explanatory variable.

Model 2: with types of diabetes-discordant comorbidities as explanatory variables.

With respect to specific types of diabetes-discordant comorbidities, depression, any lower respiratory disease, any musculoskeletal disease and severe hearing impairment were significantly and positively associated with severity of diabetes (Table 4, model 2). No significant interactions with sex were observed (data not shown in Table 4). Considering the different pathophysiological mechanisms underlying the four conditions grouped as musculoskeletal diseases, we also investigated the effect of each condition separately. All four conditions except for osteoporosis were significantly and positively associated with the grade of diabetes severity (data not shown).

## Discussion

The present study provides nationally representative data on the prevalence and comorbidity of known diabetes among older non-institutionalized adults in Germany. The overall 12 month-prevalence of known diabetes was high at 13.6% with no difference between men and women. Although we did not collect information about the type of diabetes, it can be generally assumed that the vast majority of diabetes identified from older adults aged over 50 years were type-2 diabetes. Persons with known diabetes were significantly more likely to suffer from additional chronic health conditions than persons without diabetes, irrespective of potential confounders. As expected, these associations were most pronounced for diabetes-concordant comorbidities, i. e. conditions in the pathophysiological pathway of diabetes. Severity of diabetes classified as grade 1–4 according to the type and number of concordant comorbidities positively and independently correlated with age and the number of diabetes-discordant comorbidities as well as with specific diabetes-discordant conditions including depression, chronic lower respiratory disease, musculoskeletal disease, and severe hearing impairment.

### Prevalence of known diabetes

The prevalence of diabetes varies considerably between countries [29] and even between regions within a given country [30]. This may be partly explained by differences in ethnic or socioeconomic background as well as differences in health care systems [31]. However, comparisons between studies are often compromised by methodological differences regarding data collection mode, the age range of the study population, and the diagnostic criteria used to define diabetes. In the present study, we used 12-month-prevalence estimates. Previous sex and age stratified analysis of GEDA 2009 data including study participants of all age groups showed that lifetime prevalence estimates were consistently higher than 12-month-prevalence estimates with absolute overall differences of 1.8% among women and 1.0% among men [22]. The largest differences were found among women in the age group 30–44 (2.5%) and 65+ years (2.7%) [22], probably reflecting gestational diabetes.

In Germany, several nationwide health surveys have been conducted since the reunification using standardized computer-assisted interview technique, either via telephone or face-to-face interview [11,32], Heidemann C, Du Y, Schubert I, Rathmann W, Scheidt-Nave C: Prevalence and temporal trend of known diabetes mellitus. Results of the German Health Interview and Examination Survey for Adults (DEGS1). Bundesgesundheitsblatt Gesundheitsforschung Gesundheitsschutz 2013. Forthcoming]. In these previous surveys, age-specific prevalence estimates in older age groups are similar to those observed in the present study [11]. In contrast, prevalence estimates obtained from health insurance claim data [14,15] have been consistently higher than results of national health surveys. This has partly been explained by selection bias, as most analyses are derived from AOK (Allgemeine Ortskrankenkasse) data. The AOK is a large German sickness fund insuring a particularly high proportion of older persons, persons with multiple concurrent health problems and persons on social welfare. Information on health insurance provider was obtained in GEDA, which permitted the calculation of diabetes prevalence estimates stratified by insurance company. Persons insured by the AOK were significantly more likely to have known diabetes compared to those insured by other providers (data not shown). This was also demonstrated in previous national health interview surveys conducted by telephone or postal questionnaires as well as in recent national health examination surveys based on two-stage stratified random sampling from local population registries [16,32], Heidemann C, Du Y, Schubert I, Rathmann W, Scheidt-Nave C: Prevalence and temporal trend of known diabetes mellitus. Results of the German Health Interview and Examination Survey for Adults (DEGS1). Bundesgesundheitsblatt Gesundheitsforschung Gesundheitsschutz 2013. Forthcoming]. The prevalence estimates of known diabetes among persons with AOK insurance observed in our study were similar to published prevalence estimates based on data from the AOK Hesse [15]. A recent pooled analysis of several regional population-based studies demonstrated considerable regional differences in the prevalence of known type-2 diabetes; in agreement with our results, prevalence estimates were highest in the eastern parts of Germany [12]. The underlying reasons are subject to ongoing investigation (http://www.kompetenznetz-diabetes-mellitus.net). Extending comparisons to results of national health surveys in other western countries, our recalculated age and sex-specific prevalence estimates were comparable to those reported in the US National Health and Nutrition Examination Survey 2003–2006 [33] and in the French Nutrition and Health Survey 2006–2007 [34] among persons 50–74 years of age. Among persons 75 years of age and above, our prevalence estimates exceed those obtained in the US study [33] and in the annual Health Survey for England 1994–2006 [35]. However, differences in the definition of known diabetes need to be considered, e. g. lifetime vs. 12-month prevalence estimates and exclusions of women with gestational diabetes.

### Associations between diabetes and other chronic conditions

In the present study, diabetes was significantly associated with a wide range of chronic conditions. The strength of these associations was most pronounced for diabetes-concordant comorbidities, including cardiometabolic risk factors, cardiovascular disease and chronic renal disease. These conditions are well known to be in the pathophysiological pathway of diabetes [36]. Self-reported severe visual impairment was not significantly related to diabetes in our study after adjusting for age and other covariables. There are several possible explanations for this. First, visual impairment is strongly related to older age, causes other than diabetic retinopathy prevail in older compared to younger persons with diabetes [37-40]. Thus, the difference between persons with and without diabetes may be less pronounced in older age. Secondly, self-reported visual impairment does not permit to differentiate between uncorrectable and correctable visual impairment. Uncorrectable but not correctable visual impairment as assessed by measurements of visual acuity and automated refraction was significantly more prevalent among adults with than without diabetes in the National Health Interview and Examination Survey (NHANES) 1999–2004 after adjustment for confounders [37]. Finally, the risk of severe visual impairment due to microvascular complications increases with the duration of diabetes [40] and long-standing diabetes may be underrepresented in our survey.

We also observed significant associations between diabetes and diabetes-discordant comorbidities, i. e. comorbidities less evidently related to diabetes. Previous studies reported associations of diabetes with chronic liver disease [41] and various types of cancer [42]. Study results regarding the relation between diabetes and musculoskeletal conditions are less consistent [43,44]. A relation between diabetes and certain site-specific cancers has been attributed to hyperinsulinemia, but the causal link is still subject to debate [45]. We observed no independent association between diabetes and asthma or chronic bronchitis. Results of previous investigations of these associations are conflicting [46,47]. The association of diabetes with upper gastrointestinal tract disease may imply helicobacter pylori infection as a possible common pathway of the two diseases [48]. Unlike previous studies, we did not observe a significant association between a history of diabetes and self-reported severe hearing impairment [49]. Also in contrast with earlier reports [50], an association between diabetes and depression was restricted to women in the present study. We cannot exclude that associations of diabetes with both conditions were biased towards the null in the present study due to non-participation.

### Comorbidity patterns and severity of disease among diabetics

One of the key goals of diabetes management programs is to prevent diabetes-concordant complications involving target organ damage. In our study, 43% of persons with known diabetes reported at least one such comorbidity. Few previous studies have systematically analyzed comorbidity patterns in population-based samples of persons with diabetes. In the German DETECT study of diabetic patients recruited from a nationwide sample of general practices, half of patients with type-2 diabetes (50.2%) had at least one diabetes-related micro- or macrovascular complication [17]. Similar results were also found in other studies conducted in Germany [18,51]. In a population of patients with diabetes identified from South Glamorgan in the UK, Morgan et al. found that 25.2%, 9.6%, 18.1%, 16.5% and 2.0% of diabetic patients had CHD, cerebrovascular disease, diabetic foot, retinopathy and nephropathy, respectively, while 52% of diabetic patients had none of these studied micro- and macro-vascular complications [52]. These results have been confirmed by additional regional investigations in the UK [53]. In summary, results from previous studies regarding the proportion of persons with diabetes who also have severe diabetes-related end-organ disease are roughly in line with our observations. Direct comparisons between studies are precluded by differences in study design, setting, mode of data collection as well as the type of diabetes-related comorbidities considered.

Within the given limits of the available database, we classified diabetes severity based on the type and number of diabetes-concordant comorbidities. The grading system presented here is self-developed based on evidence derived from published studies of comorbidity. It is well known that treatment and prognosis of persons with diabetes mellitus or any other index disease is likely to be influenced by co-existing health conditions, whether they are in the pathophysiologic pathway of the index disease or not [26]. This is particularly true among older persons with diabetes who tend to have multiple health conditions. In order to describe diabetes comorbidity in a comprehensive and systematic way, we applied the idea of the Cumulative Illness Rating Scale (CIRS), aggregating individual health problems according to organ systems [54,55]. From a public health perspective, it seemed crucial to develop a grading system of diabetes comorbidity that would not only permit to differentiate between diabetes-concordant and diabetes-discordant comorbidities, but also between target organ complications of diabetes and systemic cardiometabolic conditions likely to coexist or even to precede the onset of diabetes, such as hyperlipidemia. The proportion of persons with diabetes mellitus who already have macro- or microvascular complications may serve as an indicator to monitor time trends and spatial distributions of diabetes management and quality of care.

While we think this is a step in the right direction, criteria of diabetes severity in population-based epidemiological studies need to be refined and consented to be clinically relevant and to permit comparisons between studies. In particular, there is need for studies including objective measures of concomitant cardiometabolic risk factors (e. g. blood pressure, serum lipids), glucose control (glycosylated hemoglobin), inflammation (high sensitivity C-reactive protein) and diabetes-related complications (neuropathy, diabetic foot, diabetic retinopathy, diabetic nephropathy). In addition, characteristics of patient complexity (living alone, depressive symptoms, cognitive impairment) need to be considered. In the present study, we observed a significant and independent association between diabetes severity and depression, chronic lower respiratory disease, musculoskeletal disease, and severe hearing impairment. These results may indicate an interactive effect of diabetes and other chronic conditions on cardiovascular target organ damage. In fact, results from epidemiological studies have suggested that CVD may be related to depression [56], chronic obstructive pulmonary disease [57], osteoarthritis [58], and rheumatoid arthritis [59]. Further, all these conditions tend to co-exist with CVD in older individuals and may hence interfere with effective patient counseling and treatment [60]. Hearing impairment may interfere with the severity of diabetes. In a retrospective analysis of laboratory and audiometric data of diabetic patients, progression of diabetes correlated with worsening of hearing ability [61].

### Strengths and limitations

GEDA 2009 is a nationally representative recent health telephone survey with a large sample size and comprehensive information on self-reported physician-diagnosed chronic conditions. We assessed systematically concordant and discordant comorbidities of diabetes. However, there are several limitations.

First and most importantly, we have to consider selection bias. This national health survey is confined to the non-institutionalized population. Regarding the population in private households, selection bias due to the exclusion of persons residing in households without landline telephones is possible. There is evidence that the proportion of persons exclusively using a mobile phone is increasing, particularly among younger adults living on their own [62]. As the present analysis was confined to persons aged 50 years and older, selective underrepresentation of this particular subgroup can be expected to be small. However, given a cooperation rate of 51.2% at the respondent level, we cannot exclude selection bias due to other reasons. Survey adjustment weights were applied as computed from deviations between study participants and census data for the non-institutionalized German population within strata of age, sex, educational attainment, and region (see Additional file 1 and Additional file 2). Nevertheless, non-responders may well differ from study participants with respect to other characteristics relevant to the study variables of major interest and these differences may have biased our results. Data regarding diabetes-related risk factors based on census data are scarce and limited to smoking and BMI computed from self-reported body weight and height. Comparing age specific prevalence estimates of smoking and BMI status obtained in the present study to estimates derived from the Microcensus 2009 did not demonstrate significant underestimation of these risk factors (see Additional file 3).

Secondly, GEDA 2009 was not specifically designed for the investigation of diabetes and its complications, hence information on treatment, type or duration of diabetes was not collected. Type 2 diabetes can be assumed to predominate in the adult population. However, information on duration of diabetes and metabolic control would have been useful to test the hypothesis that these factors are related to higher comorbidity. This should be addressed in future studies of diabetes comorbidity. Third, the definition of some chronic conditions was rather crude. For example, we asked survey participants about any cancer without differentiation between specific types of cancer. However, some cancers are more common in patients with diabetes, while prostate cancer occurs less often in men with diabetes compared to those without diabetes [45,63]. Furthermore, some of the disease categories such as ‘musculoskeletal disease’ probably included pathogenetically heterogeneous conditions.

Finally, information on diabetes and all other 20 chronic health conditions was self-reported and verification by medical records and/or laboratory tests was not possible. Assessment of health conditions by self-report bears the risk of misclassification due to over- or underreporting. Persons with diabetes may be more likely to report asymptomatic diabetes-related health conditions such as hypertension and hyperlipidemia than persons without a diagnosis of diabetes, due to higher health care services utilization [64]. On the other hand, survey participants, in particular older persons may be unable to name or to memorize medical diagnoses correctly. A validation of self-reported diagnoses against objective health data (e. g. biochemical measurements and current medication use) in the Utrecht Health Project demonstrated that assessment by self-report is likely to lead to underestimation of disease prevalence estimates. The magnitude of bias varies according to the type of disease as well as the population studied [65]. Nevertheless, our prevalence estimates for diabetes and other highly prevalent health conditions are well in line with estimates from other populations-based studies [33,34] as well as one recent German study conducted in the primary care setting [21]. The validity of our indicator for diabetes is further supported by the fact that associations of known diabetes to sociodemographic variables and major established risk factors of diabetes were all highly significant and in the expected directions (Table 1).

## Conclusions

Among non-institutionalized German adults 50 years of age and older, the 12-month prevalence of persons with known diabetes is high and does not significantly differ between men and women. Diabetes is significantly associated with a wide spectrum of chronic comorbidities, including conditions that are directly in the pathway of diabetes as well as others that may indirectly contribute to adverse diabetes outcomes. Among persons with known diabetes, 43.0% had at least one condition likely to represent micro- or macrovascular complications of diabetes; another 46.8% had at least one out of two major cardiometabolic risk factors known to be pathophysiologically related to diabetes (hypertension, hyperlipidemia). Although these diabetes-concordant comorbidities may largely contribute to the disease burden among diabetics, the number and the type of diabetes-discordant comorbidities seem to matter as well. In particular, depression, lower respiratory disease and severe hearing impairment were closely associated with severity of diabetes defined by the pattern of diabetes-concordant comorbidities. In conclusion, diabetes-concordant as well as diabetes-discordant comorbidities need to be considered for monitoring of disease burden and quality of diabetes care in population-based epidemiological studies. Definitional standards need to be refined and consented to permit comparisons between studies and analyses of time trends and spatial distributions.

## Competing interests

The authors declared that they have no competing interests.

## Authors’ contributions

YD assisted in study design, conducted statistical analysis and drafted the manuscript. CH reviewed the manuscript critically and contributed substantially to the interpretation of study results. AG and PS reviewed the manuscript and contributed to the discussion. CSN conceptualized and supervised the study, reviewed the manuscript critically, contributed substantially to the writing of the manuscript and the interpretation of study results, and takes full responsibility for the work. All authors read and approved the final manuscript.

## Pre-publication history

The pre-publication history for this paper can be accessed here:

http://www.biomedcentral.com/1471-2458/13/166/prepub

## Supplementary Material

Additional file 1 National Telephone Health Interview Survey ‘German Health Update (GEDA)’ 2009 – Unweighted and weighted distribution of demographic characteristics as percentages in comparison with German census data. Click here for file

Additional file 2 National Telephone Health Interview Survey ‘German Health Update (GEDA)’ 2009 – Sex and age specific unweighted and weighted distribution of educational attainment (ISCED classification) as percentages in comparison with German census data. Click here for file

Additional file 3 **National Telephone Health Interview Survey ‘German Health Update (GEDA)’ 2009 – Sex and age specific unweighted and weighted prevalence of obesity (body mass index > =30 kg/m**^**2**^**) and current smoking among persons 50 years of age and older in comparison with German census data.** Click here for file
