# Supplementary material for: Prevalence and comorbidity of diabetes mellitus among non-institutionalized older adults in Germany - results of the national telephone health interview survey ‘German Health Update (GEDA)’ 2009
Source: BMC Public Health. 2013 Feb 23;13:166. doi: 10.1186/1471-2458-13-166 (PMC3599814; doi:10.1186/1471-2458-13-166)
Supplement: Additional file 1 — National Telephone Health Interview Survey ‘German Health Update (GEDA)’ 2009 – Unweighted and weighted distribution of demographic characteristics as percentages in comparison with German census data. [file 1471-2458-13-166-S1.pdf]

Additional files

Additional file 1

National Telephone Health Interview Survey 'German Health Update (GEDA)' 2009 - Unweighted and weighted distribution of demographic characteristics as percentages in comparison with German census data*

Characteristic (%)	GEDA 2009 (Unweighted)	GEDA 2009 (Weighted)	Microcensus 2007*
**Sex**			
Men	43.0	48.5	48.5
Women	57.0	51.5	51.5
**Age group (years)**			
18-24	11.4	10.0	10.0
25-39	22.4	23.0	23.0
40-49	23.2	20.3	20.3
50-59	17.4	16.2	16.2
60-69	14.2	14.0	14.0
70+	11.4	16.4	16.4
**Federal states**			
Schleswig-Holstein	3.6	3.4	3.4
Hamburg	2.3	2.2	2.2
Niedersachsen	10.1	9.5	9.5
Bremen	0.9	0.8	0.8
Nordrhein-Westfalen	23.2	21.6	21.6
Hessen	7.6	7.4	7.4
Rheinland-Pfalz	5.1	4.9	4.9
Baden-Württemberg	11.8	12.9	12.9
Bayern	14.9	15.0	15.1
Saarland	1.4	1.3	1.3
Berlin	4.7	4.3	4.3
Brandenburg	3.2	3.2	3.2
Mecklenburg-Vorpommern	1.7	2.1	2.1
Sachsen	4.5	5.4	5.4
Sachsen-Anhalt	2.4	3.1	3.1
Thüringen	2.7	2.9	2.9

* Source: Microcensus 2007; Statistisches Bundesamt, Wiesbaden (https://www.destatis.de/DE/Startseite.html)
