# Supplementary material for: Prevalence and comorbidity of diabetes mellitus among non-institutionalized older adults in Germany - results of the national telephone health interview survey ‘German Health Update (GEDA)’ 2009
Source: BMC Public Health. 2013 Feb 23;13:166. doi: 10.1186/1471-2458-13-166 (PMC3599814; doi:10.1186/1471-2458-13-166)
Supplement: Additional file 2 — National Telephone Health Interview Survey ‘German Health Update (GEDA)’ 2009 – Sex and age specific unweighted and weighted distribution of educational attainment (ISCED classification) as percentages in comparison with German census data. [file 1471-2458-13-166-S2.pdf]

Additional file 2

National Telephone Health Interview Survey 'German Health Update (GEDA)' 2009 – Sex and age specific unweighted and weighted distribution of educational attainment (ISCED classification) as percentages in comparison with German census data*

		GEDA2009 (Unweighted)			GEDA2009 (Weighted)			Microcensus 2007*		
	**Age group (years)**	**Primary**	**Middle**	**High**	**Primary**	**Middle**	**High**	**Primary**	**Middle**	**High**
**Men**										
	**18-24**	36.3	59.7	4.0	46.5	51.7	1.9	46.4	47.9	5.6
	**25-39**	4.0	53.6	42.4	13.4	59.9	26.7	13.4	52.2	34.4
	**40-49**	3.2	42.6	54.2	12.1	56.7	31.2	12.1	52.7	35.2
	**50-59**	3.3	43.1	53.6	11.8	56.4	31.8	11.8	54.4	33.9
	**60-69**	2.3	37.5	60.3	11.0	57.4	31.6	14.1	54.5	31.4
	**70+**	4.4	39.8	55.8	17.3	56.6	26.1	19.2	54.5	26.3
**Women**										
	**18-24**	36.6	58.8	4.6	42.0	54.9	3.2	42.0	48.6	9.4
	**25-39**	5.5	56.8	37.7	15.9	58.7	25.4	15.8	48.6	35.6
	**40-49**	4.9	54.1	41.0	16.4	60.5	23.1	16.3	54.3	29.4
	**50-59**	7.5	51.9	40.6	21.7	58.2	20.2	21.6	55.9	22.5
	**60-69**	10.5	56.8	32.8	32.9	54.0	13.1	33.0	52.6	14.4
	**70+**	24.0	54.1	21.9	55.3	38.2	6.5	55.2	37.2	7.6

* Source: Microcensus 2007; Statistisches Bundesamt, Wiesbaden (https://www.destatis.de/DE/Startseite.html)

ISCED: International Standard Classification of Education
