# Supplementary material for: Prevalence and comorbidity of diabetes mellitus among non-institutionalized older adults in Germany - results of the national telephone health interview survey ‘German Health Update (GEDA)’ 2009
Source: BMC Public Health. 2013 Feb 23;13:166. doi: 10.1186/1471-2458-13-166 (PMC3599814; doi:10.1186/1471-2458-13-166)
Supplement: Additional file 3 — National Telephone Health Interview Survey ‘German Health Update (GEDA)’ 2009 – Sex and age specific unweighted and weighted prevalence of obesity (body mass index > =30 kg/m2) and current smoking among persons 50 years of age and older in comparison with German census data. [file 1471-2458-13-166-S3.pdf]

Additional file 3

National Telephone Health Interview Survey 'German Health Update (GEDA)' 2009 - Sex and age specific unweighted and weighted prevalence of obesity (body mass index > =30 kg/m^2) and current smoking among persons 50 years of age and older in comparison with German census data*

		Prevalence of obesity (%)			Prevalence of current smoking (%)		
	Age group (years)	GEDA 2009 (Unweighted)	GEDA 2009 (Weighted)	Microcensus 2009*	GEDA 2009 (Unweighted)	GEDA 2009 (Weighted)	Microcensus 2009*
**Men**							
	**50-59**	19.8	23.4	20.3	33.2	34.1	33.8
	**60-69**	21.0	23.2	21.8	20.8	21.5	20.6
	**70+**	16.3	20.1	18.1	12.0	12.5	10.1
**Women**							
	**50-59**	17.4	20.5	17.1	28.0	30.0	25.3
	**60-69**	18.3	23.1	19.5	15.7	14.4	13.6
	**70+**	18.1	21.2	18.5	7.7	7.1	4.7

* Source: Microcensus 2009; Statistisches Bundesamt, Wiesbaden 2011

https://www.destatis.de/DE/Publikationen/Thematisch/Gesundheit/Gesundheitszustand/Rauchgewohnheiten.html

https://www.destatis.de/DE/Publikationen/Thematisch/Gesundheit/Gesundheitszustand/Koerpermasse.html
